# Bioactive Constituents from the Whole Plants of *Gentianella acuta* (Michx.) Hulten

**DOI:** 10.3390/molecules22081309

**Published:** 2017-08-06

**Authors:** Zhijuan Ding, Yanxia Liu, Jingya Ruan, Shengcai Yang, Haiyang Yu, Meiling Chen, Yi Zhang, Tao Wang

**Affiliations:** 1 Tianjin State Key Laboratory of Modern Chinese Medicine, 312 Anshanxi Road, Nankai District, Tianjin 300193, China; 15222792071@163.com (Z.D.); liuyanxia210@163.com (Y.L.); Ruanjy19930919@163.com (J.R.); 15122473723@163.com (S.Y.); yuhaiyang19830116@hotmail.com (H.Y.); custard.chen@tjutcm.edu.cn (M.C.); 2 Tianjin Key Laboratory of TCM Chemistry and Analysis, Institute of Traditional Chinese Medicine, Tianjin University of Traditional Chinese Medicine, 312 Anshanxi Road, Nankai District, Tianjin 300193, China

**Keywords:** *Gentianella acuta (Michx.)* Hulten, lignan, iridoid- and secoiridoid-type monoterpene, intestine motility, mouse isolated intestine tissue

## Abstract

As a Mongolian native medicine and Ewenki folk medicinal plant, *Gentianella acuta* has been widely used for the treatment of diarrhea, hepatitis, arrhythmia, and coronary heart disease. In the course of investigating efficacy compounds to treat diarrhea using a mouse isolated intestine tissue model, we found 70% EtOH extract of *G. acuta* whole plants had an inhibitory effect on intestine contraction tension. Here, nineteen constituents, including five new compounds, named as gentiiridosides A (**1**), B (**2**), gentilignanoside A (**3**), (1*R*)-2,2,3-trimethyl-4-hydroxymethylcyclopent-3-ene-1-methyl-*O*-β-d-glucopyranoside (**4**), and (3*Z*)-3-hexene-1,5-diol 1-*O*-α-l-arabinopyranosyl(1→6)-β-d-glucopyranoside (**5**) were obtained from it. The structures of them were elucidated by chemical and spectroscopic methods. Furthermore, the inhibitory effects on motility of mouse isolated intestine tissue of the above mentioned compounds and other thirteen iridoid- and secoiridoid-type monoterpenes (**7**–**10**, **13**–**16**, **18**, **19**, **21**, **22**, and **25**) previously obtained in the plant were analyzed. As results, new compound **5**, some secoiridoid-type monoterpenes **7**, **10**, **12**–**14**, **16**, and **17**, as well as 7-*O*-9′-type lignans **31** and **32** displayed significant inhibitory effect on contraction tension at 40 μM.

## 1. Introduction

**Figure 1:**
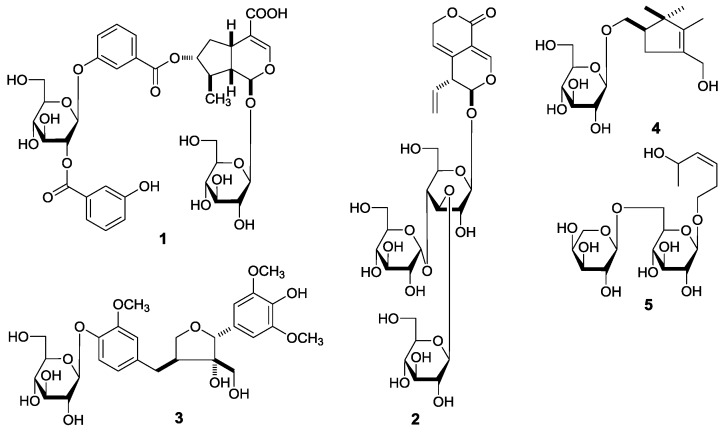
The new compounds **1**–**5** obtained from the whole plant of *G. acuta*.

**Figure 2:**
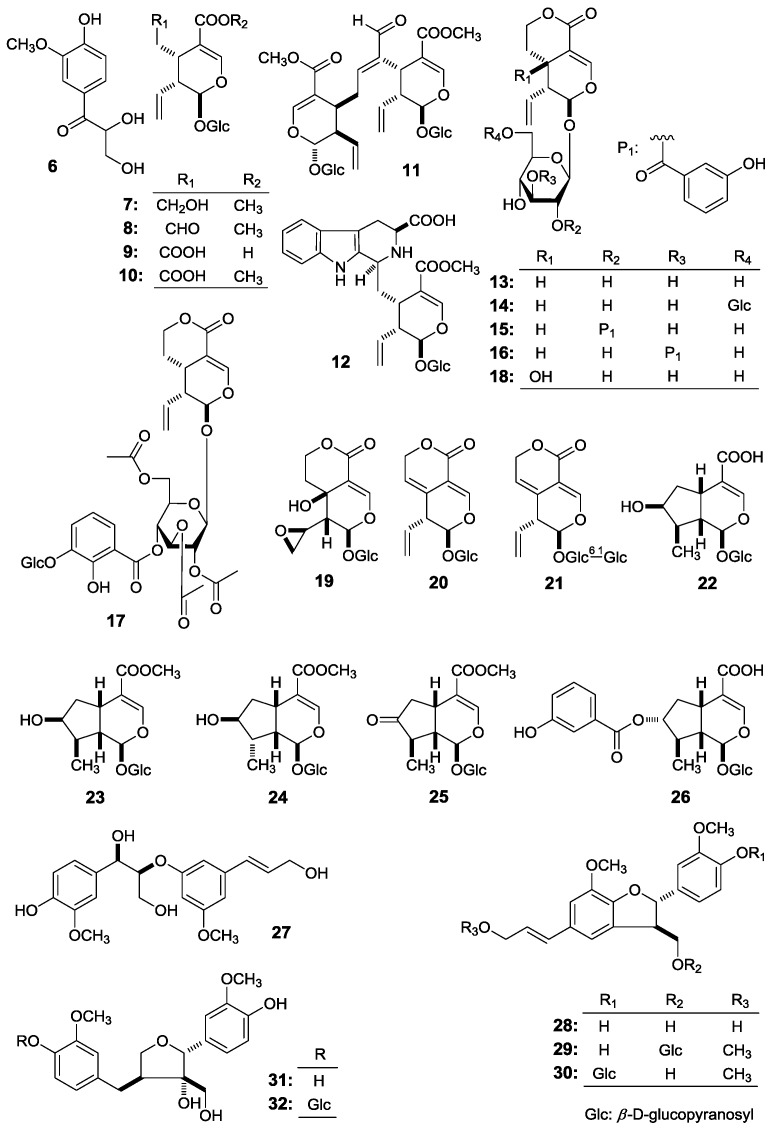
The known compounds (**6**–**32**) obtained from the whole plant of *G. acuta*.

*Gentianella acuta* (Michx.) Hulten belongs to the family Gentianaceae, distributed mainly in the north of China, Mongolia plateau, Siberia, and Far East areas of Russia [1]. As a Mongolian native medicine and Ewenki folk medicinal plant, *G. acuta* has been widely used for the treatment of diarrhea, hepatitis, arrhythmia, coronary heart disease, jaundice, fever, and headache [1,2,3]. In the course of investigating efficacy compounds to treat diarrhea using a mouse isolated intestine tissue model, we found that a 70% EtOH extract of *G. acuta* whole plants had an inhibitory effect on intestine contraction tension. Moreover, eighteen xanthones had been obtained and their inhibitory effects on the model were assayed. As results, some xanthones were found to have a significant reducing effect on intestine contraction tension [4]. Additionally, for xanthones, monoterpenes, and lignans were elucidated to be main constituents in the plant in our continuing study, among them, the spectroscopy data of thirteen iridoid- and secoiridoid-type monoterpenes had been reported [5,6] by us. Here, we obtained other nineteen constituents, including five new compounds (**1**–**5**) (Figure 1) and fourteen known ones (**6**, **11**, **12**, **17**, **20**, **23**, **24**, **26**–**32**) (Figure 2). Do they have an inhibitory effect on motility of mouse isolated intestine tissue, too? In this paper, we describe the isolation and structure elucidation of them, along with evaluations of their inhibitory effects on intestine contraction tension.

## 2. Results and Discussion

During the course of our continuous studies on bioactive constituents from a 95% EtOH eluate of D101 CC and CHCl_3_ layer [4,5,6], obtained from the whole plants of *G. acuta*, nineteen constituents, including five new compounds, named as gentiiridosides A (**2**), B (**2**), gentilignanoside A (**3**), (1*R*)-2,2,3-trimethyl-4-hydroxymethylcyclopent-3-ene-1-methyl-*O*-β-d-glucopyranoside (**4**), and (3*Z*)-3-hexene-1,5-diol 1-*O*-α-l-arabinopyranosyl(1→6)-β-d-glucopyranoside (**5**) (Figure 1), together with fourteen known ones, 2,3-dihydroxy-1-(4-hydroxy-3-methoxyphenyl)-propan-1-one (**6**) [7] (*E*)-aldosecologanin (**11**) [8], 5α-carboxystrictosidine (**12**) [9], trifloroside (**17**) [10], gentiopicroside (**20**) [11], loganin (**23**) [12], 8-epiloganin (**24**) [13], swertiaside (**26**) [14], (7*R*,8*S*)-*erythro*-4,7,9,9′-tetrahydroxy-3,3′-dimethoxy-8-*O*-4′-neolignan (**27**) [15,16], (7*S*,8*R*)-dehydrodiconiferyl alcohol (**28**) [17,18,19], plucheoside D_3_ (**29**) [17,18,20], (7*S*,8*R*)-9′-methoxy-dehydrodiconiferyl alcohol 4-*O*-β-d-glucopyranoside (**30**) [17,18,21], (–)-berchemol (**31**) [22,23], and berchemol-4′-*O*-β-d-glucoside (**32**) [22,24] (Figure 2), were further obtained. Among the known isolates, **6**, **11**, **12**, **17**, **23**, and **27**–**32** were isolated from the genus firstly.

This paper will elucidate the isolation and structure of the new compounds. Meanwhile, the effects of the abovementioned compounds and previously-isolated thirteen iridoid- and secoiridoid-type monoterpenes, secologanol (**7**) [5], secologanin [6] (**8**), secologanoside (**9**) [5], secoxyloganin (**10**) [5], sweroside (**13**) [6], swertiapunimarin (**14**) [5], deacetylcentapicrin (**15**) [6], decentapicrin A (**16**) [6], swertiamarin (**18**) [5], eustomoside (**19**) [5], 6′-*O*-β-d-glucopyranosyl gentiopicroside (**21**) [5], loganic acid (**22**) [6], 7-ketologanin (**25**) [6] (Figure 2) on the motility of mouse isolated intestine tissue were determined.

**Figure 3:**
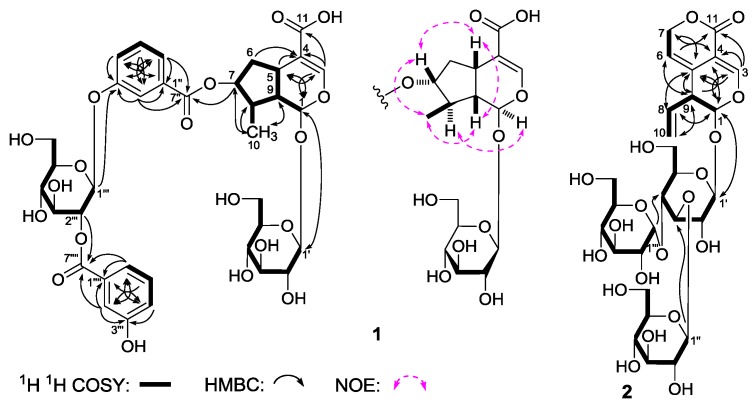
The main ^1^H ^1^H COSY, HMBC correlations of **1** and **2**, and NOE correlations of **1**.

*Gentiiridoside A* (**1**) was isolated as a white powder with negative optical rotation [[α${]}_{D}^{25}$ −90.0° (*c* 0.14, MeOH)]. Negative high resolution electrospray ionization-time of flight-mass spectra (HRESI-TOF-MS) afforded [M – H]^−^ at *m*/*z* 777.2261 (calcd for C_36_H_41_O_19_, 777.2248), supporting a molecular formula of C_36_H_42_O_19_ for **1**. The absorption bands showed in the infrared (IR) spectrum suggested the presence of hydroxyl (3372 cm^−1^), α,β-unsaturated carbonyl (1712 cm^−1^), aromatic ring (1635, 1588, 1486 cm^−^^1^), and *O*-glycosidic linkage (1078 cm^−1^). The sugars in **1** were found to be d-glucose by acid hydrolysis with 1 M HCl [4]. The ^1^H, ^13^C-nuclear magnetic resonance (NMR) spectra (Table 1) and various two-dimensional (2D) NMR spectra, including ^1^H ^1^H chemical-shift correlation spectroscopy (^1^H ^1^H COSY), heteronuclear single quantum correlation (HSQC), heteronuclear multiple bond correlation (HMBC) displayed signals assignable to two 3-hydroxy benzoyl {[δ 7.42 (1H, br. d, *ca*. *J* = 8 Hz, H-4′′), 7.48 (1H, dd, *J* = 7.5, 7.5 Hz, H-5′′), 7.84 (1H, br. d, *ca*. *J* = 8 Hz, H-6′′), 7.89 (1H, br. s, H-2′′); *δ*_C_ 166.1 (C-7′′)]; [*δ* 7.45 (1H, br. d, *ca*. *J* = 8 Hz, H-4′′′′), 7.54 (1H, dd, *J* = 7.5, 7.5 Hz, H-5′′′′), 7.82 (1H, br. s, H-2′′′′), 7.88 (1H, br. d, *ca*. *J* = 8 Hz, H-6′′′′); *δ*_C_ 166.8 (C-7′′′′)]}, and two β-d-glucopyranosyl [δ 4.69 (1H, d, *J* = 7.5 Hz, H-1′), 5.02 (1H, d, *J* = 8.0 Hz, H-1′′′)]. On the other hand, thirty-six carbon signals were shown in its ^13^C-NMR spectrum, in addition to twenty-six carbon signals occupied by the abovementioned fragments, the other 10 carbon signals, together with the relative proton signals [δ 5.50 (1H, d, *J* = 3.0 Hz, H-1), 7.46 (1H, s, H-3)] indicated the aglycon of **1** was iridoid. As shown in Figure 3, the ^1^H ^1^H COSY experiment on **1** suggested the existence of five partial structures. Furthermore, in the HMBC experiment, long-range correlations from δ_H_ 5.50 (H-1) to δ_C_ 152.4 (C-3); δ_H_ 7.46 (H-3) to δ_C_ 32.5 (C-5), 96.2 (C-1), 112.8 (C-4), 170.9 (C-11); δ_H_ 4.96 (H-7) to δ_C_ 166.1 (C-7′′); δ_H_ 4.69 (H-1′) to δ_C_ 96.2 (C-1); δ_H_ 7.89 (H-2′′), 7.84 (H-6′′) to δ_C_ 166.1 (C-7′′); δ_H_ 5.02 (H-1′′′) to δ_C_ 159.2 (C-3′′); δ_H_ 3.51 (H-2′′′) to δ_C_ 166.8 (C-7′′′′); δ_H_ 7.82 (H-2′′′′), 7.88 (H-6′′′′) to δ_C_ 166.8 (C-7′′′′) were observed. Therefore, the planar structure of **1** was constructed. The relative configuration of **1** was determined by a nuclear Overhauser effect spectroscopy (NOESY) experiment, and NOE correlations were observed between H-1 and H-8; H-5 and H-7; H_3_-10 and H-7, H-9. The ^1^H and ^13^C-NMR sepctra of **1** were found very similar to those of swertiaside (**26**) [14], except that a 2-(3-hydroxybenzoyl)-β-d-glucopyranosyl appeared at the 3′′-position in **1**. Consequently, the structure of gentiiridoside A (**1**) was determined.

*Gentiiridoside B* (**2**) was obtained as a white powder with negative optical rotation [[α${]}_{D}^{25}$ −36.7° (*c* 0.12, MeOH)]. HRESI-TOF-MS exhibited a molecular ion peak at *m/z* 679.1023 [M – H]^−^, and revealed the molecular formula C_28_H_40_O_18_ (calcd for C_28_H_39_O_18_, 679.1033) for it. Acid hydrolysis of **2** with 1 M HCl afforded d-glucose, whose absolute configuration was determined by HPLC analysis [4]. The ^1^H and ^13^C-NMR (Table 2) spectra of **2** indicated the presence of two β-d-glucopyranosyl [δ 4.27 (1H, d, *J* = 7.5 Hz, H-1′′), 4.49 (1H, d, *J* = 8.0 Hz, H-1′)], and one α-d-glucopyranosyl [δ 4.91 (1H, d, *J* = 3.5 Hz, H-1′′′)]. Twenty-eight carbon signals were displayed in its ^13^C-NMR spectrum, except for the above mentiond moieties, the other ten signals as well as their relative ^1^H-NMR signals [δ 5.21 (2H, m, H_2_-10), 5.59 (1H, d, *J* = 3.0 Hz, H-1), 5.72 (1H, ddd, *J* = 6.5, 10.5, 17.5 Hz, H-8), 7.41 (1H, s, H-3)] suggested the aglycon of **2** was the same as that of gentiopicroside (**20**) [11]. Finally, the long-range correlations from δ_H_ 5.59 (H-1) to δ_C_ 124.9 (C-5), 148.8 (C-3); δ_H_ 7.41 (H-3) to δ_C_ 96.4 (C-1), 103.2 (C-4), 124.9 (C-5), 162.7 (C-11); δ_H_ 5.65 (H-6) to δ_C_ 44.3 (C-9), 103.2 (C-4); δ_H_ 4.97, 5.04 (H_2_-7) to δ_C_ 124.9 (C-5), 162.7 (C-11); δ_H_ 5.72 (H-8) to δ_C_ 96.4 (C-1), 124.9 (C-5); δ_H_ 3.31 (H-9) to δ_C_ 103.2 (C-4), 116.1 (C-6), 124.9 (C-5); δ_H_ 4.49 (H-1′) to δ_C_ 96.4 (C-1); δ_H_ 4.27 (H-1′′) to δ_C_ 76.7 (C-1′); δ_H_ 4.91 (H-1′′′) to δ_C_ 69.9 (C-1′′) were observed in the HMBC spectrum. Then, the structure of gentiiridoside B (**2**) was clarified.

**Figure 4:**
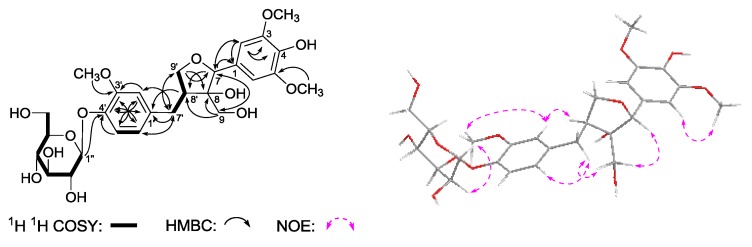
The main ^1^H ^1^H COSY, HMBC, and NOE correlations of **3**.

**Figure 5:**
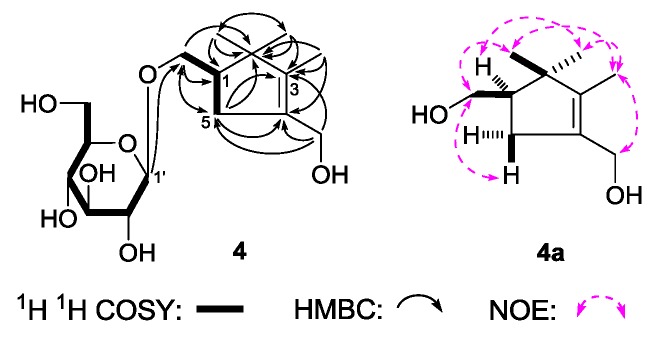
The main ^1^H ^1^H COSY, HMBC correlations of **4** and **4a**.

**Figure 6:**
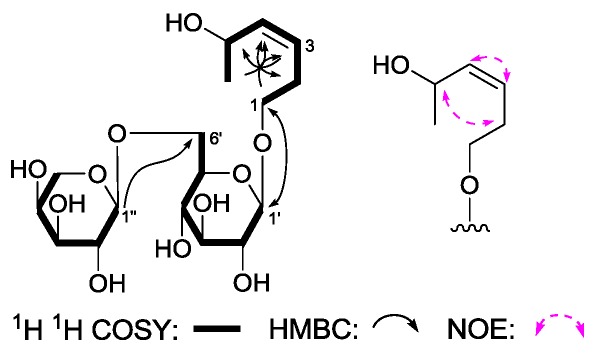
The main ^1^H ^1^H COSY, HMBC, and NOE correlations of **5**.

*Gentilignanoside A* (**3**) was obtained as a white powder that exhibited negative optical rotation [[α${]}_{D}^{25}$ −36.0° (*c* 0.10, MeOH)]. The molecular formula, C_27_H_34_O_13_, of **3** was determined from Q-TOF-ESI-MS analysis (*m*/*z* 567.2083 [M – H]^−^, calcd for C_27_H_33_O_13_, 567.2083). Its IR spectrum showed absorption bands due to hydroxyl (3368 cm^−1^), aromatic ring (1613, 1513, 1463 cm^−1^), and *O*-glycosidic linkage (1073 cm^−1^). The ^1^H, ^13^C-NMR spectra (Table 3) and kinds of 2D NMR spectra (^1^H ^1^H COSY, HSQC, HMBC) showed signals ascribable to one ABX-type aromatic protons [δ 6.77 (1H, br. d, *ca. J* = 8 Hz, H-6′), 6.90 (1H, br. s, H-2′), 7.10 (1H, d, *J* = 8.0 Hz, H-5′)], one 1,3,4,5-symmetrical substituted phenyl group [δ 6.64 (2H, s, H-2,6)], two methylene bearing oxygen funtion {δ [3.64, 3.80 (1H each, both m, overlapped, H_2_-9)], [3.64 (1H, m, overlapped), 4.06 (1H, dd, J = 7.5, 7.5 Hz), H_2_-9′]]}, three methoxyl [δ 3.84 (6H, s, 3,5-OCH_3_), 3.86 (3H, s, 3′-OCH_3_)], and one β-d-glucopyranosyl [δ 4.88 (1H, d, *J* = 7.5 Hz, H-1′′)]. The planar structure of **3** was constructed by the assignment of the ^1^H ^1^H COSY and HMBC experiments as shown in Figure 4. The ^1^H ^1^H COSY experiment indicated the presence of three partial moieties. On the other hand, in the HMBC experiment, long-range correlations were found from the following proton and carbon pairs: δ_H_ 6.64 (H-2,6) to δ_C_ 129.9 (C-1), 136.2 (C-4), 148.9 (C-3,5); δ_H_ 4.84 (H-7) to δ_C_ 51.8 (C-8′), 64.6 (C-9), 106.2 (C-2,6), 129.9 (C-1); δ_H_ 3.64, 3.80 (H_2_-9) to δ_C_ 51.8 (C-8′), 83.3 (C-8), 85.8 (C-7); δ_H_ 6.90 (H-2′) to δ_C_ 35.1 (C-7′), 122.4 (C-6′), 146.5 (C-4′), 150.9 (C-3′); δ_H_ 7.10 (H-5′) to δ_C_ 136.9 (C-1′), 146.5 (C-4′), 150.9 (C-3′); δ_H_ 6.77 (H-6′) to δ_C_ 35.1 (C-7′), 114.4 (C-2′), 146.5 (C-4′); δ_H_ 2.54, 3.13 (H_2_-7′) to δ_C_ 72.0 (C-9′), 114.4 (C-2′), 122.4 (C-6′), 136.9 (C-1′); δ_H_ 2.59 (H-8′) to δ_C_ 136.9 (C-1′); δ_H_ 3.64, 4.06 (H_2_-9′) to δ_C_ 35.1 (C-7′), 83.3 (C-8), 85.8 (C-7); δ_H_ 3.84 (3,5-OCH_3_) to δ_C_ 148.9 (C-3,5); δ_H_ 3.86 (3′-OCH_3_) to δ_C_ 150.9 (C-3′); δ_H_ 4.88 (H-1′′) to δ_C_ 146.5 (C-4′). Furthermore, the relative configuration of **3** was determined by the NOE correlations between δ_H_ 4.84 (H-7) and δ_H_ 3.64, 3.80 (H_2_-9); δ_H_ 3.64, 3.80 (H_2_-9) and δ_H_ 2.54, 3.13 (H_2_-7′) observed in its NOESY spectrum. Finally, **3** showed negative Cotton effect at 278 and 232 nm, which indicated the absolute configuration of it was 7*R*,8*S*,8′*S* [22].

(1R)-2,2,3-Trimethyl-4-hydroxymethylcyclopent-3-ene-1-methyl-O-β-d-glucopyranoside (**4**), was obtained as a white powder. It had the molecular formula C_16_H_28_O_7_, determined by negative-ion HRESI-TOF-MS (*m/z* 377.1814 [M + COOH]^−^, calcd for C_17_H_29_O_9_, 377.1817). Its IR spectrum showed absorption bands at 3367, 1636, and 1076 cm^−1^ ascribable to hydroxyl, olefin, and *O*-glycosidic linkages, respectively. It was reated with 1 M HCl to give d-glucose [4]. The ^1^H, ^13^C-NMR (Table 4) spectra showed signals assignable to three methyl [δ 0.87, 1.09, 1.56 (3H each, all s, 2β, 2α, 3-CH_3_)], two methylene with oxygen function {[δ 3.66 (1H, m, overlapped), 3.95 (1H, dd, *J* = 6.5, 11.0 Hz), 1-C*H*_2_OH], 4.07 (2H, d, *J* = 9.0 Hz, 4-C*H*_2_OH)}, one β-d-glucopyranosyl [δ 4.26 (1H, d, *J* = 7.5 Hz, H-1′)], one methylene [δ 2.09 (1H, dd, *J* = 8.0, 9.0 Hz), 2.49 (1H, dd, *J* = 8.0, 8.0 Hz), H_2_-5], together with one methine [δ 2.15 (1H, m, overlapped, H-1)]. The ^1^H ^1^H COSY experiment suggested the presence of two partial fragments shown in bold lines (Figure 5). Then, the planar structure of **4** was further elucidated by the long-range correlations from δ_H_ 0.87 (2β-CH_3_) to δ_C_ 27.2 (2α-CH_3_), 49.0 (C-1), 49.3 (C-2), 143.5 (C-3); δ_H_ 1.09 (2α-CH_3_) to δ_C_ 20.1 (2β-CH_3_), 49.0 (C-1), 49.3 (C-2), 143.5 (C-3); δ_H_ 1.56 (3-CH_3_) to δ_C_ 49.3 (C-2), 133.1 (C-4), 143.5 (C-3); δ_H_ 4.07 (4-C*H*_2_OH) to δ_C_ 36.6 (C-5), 133.1 (C-4), 143.5 (C-3); δ_H_ 2.15, 2.48 (H_2_-5) to δ_C_ 49.3 (C-2), 72.1 (1-CH_2_OH), 133.1 (C-4), 143.5 (C-3); δ_H_ 4.26 (H-1′) to δ_C_ 72.1 (1-CH_2_OH) observed in the HMBC spectrum. To determine the sterostructure of it, **4** was treated with β-glucosidase, to give the aglycon, (1*R*)-2,2,3-trimethyl-4-hydroxymethylcyclopent-3-ene-1-methanol (**4**a), which was obtained with negative optical rotation ([α]_D_ −6.5°, CHCl_3_) and had only one chiral carbon. Using the the same method reported in literatures [25,26], compared optical rotation of **4a** with that of its simialr compound, (–)-(*R*)-*γ*-necrodol ([α]_D_ −21.2°, CHCl_3_) [27], the absolute configuration of **4** was elucidated to be 1*R*. Finally, the chemical shift of two methyl at the 2-position was determined by NOE correlations displayed in NOESY experiment. On the basis of above mentioned evidences, the structure of **4** was identified as (1*R*)-2,2,3-trimethyl-4-hydroxymethylcyclopent-3-ene-1-methyl-*O*-β-d-glucopyranoside.

(3Z)-3-Hexene-1,5-diol 1-O-α-l-arabinopyranosyl(1→6)-β-d-glucopyranoside (**5**) was obtained as a white powder with negative optical rotation [[α${]}_{D}^{25}$ −14.5° (*c* 0.11, MeOH)]. Its molecular formula, C_17_H_30_O_11_ (*m*/*z* 455.1773 [M + COOH]^−^; calcd for C_18_H_31_O_13_, 455.1770), was recorded by Q-TOF-ESI-MS. Furthermore, using acid hydrolysis and HPLC analysis, the presence of d-glucose and l-arabinose in **5** was revealed [4]. The ^1^H, ^13^C-NMR spectra (Table 5) and 2D NMR (^1^H ^1^H COSY, HSQC, HMBC) spectra indicated the presence of two olefinic protons [δ 5.45, 5.47 (1H each, both m, H-3 and 4)], one methoxyl [δ 1.20 (3H, d, *J* = 6.0 Hz, H_3_-6)], one β-d-glucopyranosyl [δ 4.27 (1H, d, *J* = 7.5 Hz, H-1′)], along with one α-l-arabinopyranosyl [δ 4.31 (1H, d, *J* = 6.5 Hz, H-1′′)]. The planar structure of **5** was constructed on the basis of ^1^H ^1^H COSY and HMBC experiments. Namely, the ^1^H ^1^H COSY experiment suggested the existence of three partial structures, as shown as bold lines in Figure 6. Meanwhile, in its HMBC spectrum, long-rang correlations from δ_H_ 4.27 (H-1′) to δ_C_ 70.4 (C-1); δ_H_ 4.31 (H-1′′) to δ_C_ 69.6 (C-6′) were observed. Finally, the NOE correlation between δ_H_ 2.39, 2.46 (H_2_-2), and δ_H_ 4.61 (H-5); 5.45 (H-3) and δ_H_ 5.47 (H-5) found in the NOESY spectrum indicated the configuration in the 3-position was *Z*. Consequently, the structure of **5** was elucidated to be (3*Z*)-3-hexene-1,5-diol 1-*O*-α-l-arabinopyranosyl(1→6)-β-d-glucopyranoside.

Furthermore, inhibitory effects of all fractions obtained from 70% EtOH extract of *G. acuta* and the abovementioned isolates on motility of mouse isolated intestine tissue were determined by using the same method as reported previously [4,28]. As results, all of the test samples showed no significant changing on isolated intestinal tissue contraction frequency, while 70% EtOH extract of *G. acut*, 95% EtOH eluate from D101 macroporous resin CC, CHCl_3_ layer, as well as compounds **5**, **7**, **10**, **12**–**14**, **16**, **17**, **31**, and **32** displayed significant inhibitory effects on contraction tension (Table 6).

From the whole plants of *G. acuta*, two types of monoterpenes, iridoid- (**1**, **22**–**26**) and secoiridoid-type (**7**–**21**) monoterpenes were obtained. Structure-activity relationship analysis revealed that iridoid-type monoterpenes showed no significant effect on contraction tension. However, secoiridoid-type monoterpenes, such as **7**, **10**, **12**–**14**, **16**, **17** had strong inhibitory effect. Furthermore, when an olefin functional existed between the 5- and 6-positions (**2**, **20**, **21**), or H-5 was substituted by hydroxyl (**18**, **19**), the bioactivity disappeared.

Meanwhile, comparing the inhibitory effect on contraction tension of 8-*O*-4′- (**27**), 7-*O*-4′- (**28**–**30**) with those of 7-*O*-9′-type (**31**, **32**) lignan, we found that 7-*O*-9′-type (**31**, **32**) lignan displayed strong inhibitory bioactivity on contraction tension.

## 3. Experimental

### 3.1. General

Physical data was obtained by using the following instruments: UV and IR spectra were determined on a Varian Cary 50 UV-VIS (Varian, Inc., Hubbardsdon, MA, USA) and Varian 640-IR FT-IR spectrophotometer (Varian Australia Pty Ltd., Mulgrave, Australia), respectively. Optical rotations were obtained on a Rudolph Autopol^®^ IV automatic polarimeter (l = 50 mm) (Rudolph Research Analytical, 55 Newburgh Road, Hackettstown, NJ, 07840 USA). NMR spectra were run on a Bruker 500 MHz NMR spectrometer (Bruker BioSpin AG Industriestrasse 26 CH-8117, Fällanden, Switzerland) at 500 MHz for ^1^H and 125 MHz for ^13^CNMR (internal standard: TMS). Negative-ion HRESI-TOF-MS were recorded on an Agilent 6520 Accurate-Mass Q-Tof LC/MS spectrometer (Agilent Corp., Santa Clara, CA, USA). Column chromatographies (CC) were performed on macroporous resin D101 (Haiguang Chemical Co., Ltd., Tianjin, China), silica gel (74–149 μm, Qingdao Haiyang Chemical Co., Ltd., Qingdao, China), and Sephadex LH-20 (Ge Healthcare Bio-Sciences, Uppsala, Sweden). Preparative high-performance liquid chromatography (PHPLC) column, cosmosil 5C_18_-MS-II (20 mm i.d. × 250 mm, Nakalai Tesque, Inc., Tokyo, Japan) were used to isolate the compounds.

### 3.2. Plant Material

The whole plants of *Gentianella acuta* (Michx.) Hulten were collected from Alxa Youqi, Inner Mongolia Autonomous region, China in September 2013, and identified by Dr. Li Tianxiang (Experiment Teaching Department, Tianjin University of Traditional Chinese Medicine). The voucher specimen was deposited at the Academy of Traditional Chinese Medicine of Tianjin University of TCM.

### 3.3. Extraction and Isolation

The whole plants of *G. acuta* (3.0 kg) were cut and refluxed with 70% ethanol–water. Then the 70% EtOH extract (868.5 g) was partitioned in a CHCl_3_–H_2_O mixture (1:1, *v*/*v*). The H_2_O layer (670.0 g) was subjected to D101 macroporous resin CC (H_2_O → 95% EtOH → acetone). As a result, H_2_O (332.4 g), 95% EtOH (294.9 g), and acetone (5.1 g) eluates were obtained.

The 95% EtOH eluate (200.0 g) was subjected to silica gel CC [CHCl_3_ → CHCl_3_-MeOH (100:1 → 100:5, *v*/*v*) → CHCl_3_–MeOH–H_2_O (10:3:1 → 7:3:1 → 6:4:1, *v*/*v*/*v*, lower layer)] to give 16 fractions (Fr. 1–Fr. 16). Fraction 7 (25.7 g) was centrifugated (MeOH), and two fractions (Fr. 7-1–Fr. 7-2) were yielded. Fraction 7-2 (9.8 g) was separated by PHPLC [CH_3_CN–H_2_O (18:82 → 35:65 → 42:58, *v*/*v*) + 1% HAc] to give 27 fractions (Fr. 7-2-1–Fr. 7-2-27). Fraction 7-2-2 (70.6 mg) was purified by PHPLC [MeOH–H_2_O (22:78, *v*/*v*)], and 2,3-dihydroxy-1-(4-hydroxy-3-methoxyphenyl)-propan-1-one (**6**, 5.9 mg) was given. Fraction 7-2-8 (46.3 mg) was isolated by PHPLC [CH_3_CN-H_2_O (20:80, *v*/*v*) + 1% HAc] to gain (7*R*,8*S*)-*erythro*-4,7,9,9′-tetrahydroxy-3,3′-dimethoxy-8-*O*-4′-neolignan (**27**, 6.0 mg). Fraction 9 (15.0 g) was subjected to Sephadex LH-20 CC [CHCl_3_–MeOH (1:1, *v*/*v*)] to yield seven fractions (Fr. 9-1–Fr. 9-7). Fraction 9-4 (7.3 g) was separated by PHPLC [CH_3_CN–H_2_O (22:78 → 30:70 → 45:55, *v*/*v*) + 1% HAc], as a result, 12 fractions (Fr. 9-4-1–Fr. 9-4-12) were obtained. Fraction 9-4-2 (962.8 mg) was purified by PHPLC [MeOH–H_2_O (23:77, *v*/*v*) + 1% HAc] to give gentiiridoside B (**2**, 14.8 mg). Fraction 9-4-9 (277.1 mg) was centrifuged (MeOH), and two fractions (Fr. 9-4-9-1–Fr. 9-4-9-2) were gained. Fraction 9-4-9-2 (199.7 mg) was isolated by PHPLC [MeOH–H_2_O (45:55, *v*/*v*) + 1% HAc] to yield plucheoside D_3_ (**29**, 8.8 mg). Fraction 11 (20.0 g) was subjected to PHPLC [CH_3_CN–H_2_O (15:85 → 25:75 → 42:58, *v*/*v*) + 1% HAc], and 29 fractions (Fr. 11-1–Fr. 11-29) were given. Fraction 11-5 (631.3 mg) was separated by PHPLC [CH_3_CN–H_2_O (10:90, *v*/*v*) + 1% HAc], and 8-epiloganin (**24**, 29.7 mg) was yielded. Fraction 11-6 (388.0 mg) was isolated by PHPLC [CH_3_CN–H_2_O (11:89, *v*/*v*) + 1% HAc] to yield gentilignanoside A (**3**, 9.2 mg), loganin (**23**, 143.5 mg), and berchemol-4′-*O*-β-d-glucoside (**32**, 84.5 mg). Fraction 11-12 (660.5 mg) was centrifugated (MeOH) and further purified by PHPLC [MeOH–H_2_O (35:65, *v*/*v*) + 1% HAc] to give (1*R*)-2,2,3-trimethyl-4-hydroxymethylcyclopent-3-ene-1-methyl-*O*-β-d-glucopyranoside (**4**, 11.5 mg). Fraction 11-17 (790.7 mg) was isolated by PHPLC [MeOH–H_2_O (42:58, *v*/*v*) + 1% HAc] to obtain six fractions (Fr. 11-17-1–Fr. 11-17-6). Fraction 11-17-1 (97.1 mg) was purified by PHPLC [CH_3_CN-H_2_O (24:76, *v*/*v*) + 1% HAc] to gain swertiaside (**26**, 79.0 mg). Fraction 11-21 (376.1 mg) was separated by PHPLC [MeOH–H_2_O (42:58, *v*/*v*) + 1% HAc] to afford (7*S*,8*R*)-9′-methoxy-dehydrodiconiferyl alcohol 4-*O*-β-d-glucopyranoside (**30**, 17.4 mg). Fraction 13 (20.0 g) was subjected to PHPLC [MeOH-H_2_O (35:65 → 45:55 → 55:45, *v*/*v*) + 1% HAc], and 20 fractions (Fr. 13-1–Fr. 13-20) were yielded. Fraction 13-11 (1.9 g) was centrifuged (MeOH) to obtain two fractions (Fr. 13-11-1–Fr. 13-11-2). Fraction 13-11-1 (1.0 g) was isolated by PHPLC [CH_3_CN–H_2_O (16:84, *v*/*v*) + 1% HAc], as a result, eleven fractions (Fr. 13-11-1-1–Fr. 13-11-1-11) were given. Fraction 13-11-1-9 (117.1 mg) were purified by Sephadex LH-20 CC (MeOH) and PHPLC [MeOH–H_2_O (35:65, *v*/*v*) + 1% HAc] to afford 5α-carboxystrictosidine (**12**, 26.2 mg). Fraction 13-12 (501.4 mg) was isolated by PHPLC [CH_3_CN–H_2_O (18:82, *v*/*v*) + 1% HAc] and to yield (*E*)-aldosecologanin (**11**, 38.2 mg). Fraction 13-17 (504.0 mg) was purified by PHPLC [CH_3_CN–H_2_O (22:78, *v*/*v*) + 1% HAc] to yield gentiiridoside A (**1**, 265.0 mg). Fraction 14 (15.3 g) was subjected to PHPLC [CH_3_CN–H_2_O (15:85 → 25:75, *v*/*v*) + 1% HAc], and 20 fractions (Fr. 14-1–Fr. 14-20) were obtained. Fraction 14-1 (1.4 g) was separated by PHPLC [CH_3_CN–H_2_O (9:91, *v*/*v*) + 1% HAc] to gain 14 fractions (Fr. 14-1-1–Fr. 14-1-14). Fraction 14-1-2 (32.1 mg) was purified by Sephadex LH-20 CC (MeOH) and finally by PHPLC [CH_3_CN–H_2_O (7:93, *v*/*v*)] to give (*3Z*)-3-hexene-1,5-diol 1-*O*-α-l-arabinopyranosyl(1→6)-β-d-glucopyranoside (**5**, 2.8 mg).

The CHCl_3_ layer (50.0 g, Fr. C) was subjected to Silica gel CC [CHCl_3_–MeOH (100:2 → 100:3 → 100:5, *v*/*v*) → CHCl_3_–MeOH–H_2_O (10:3:1, *v*/*v*/*v*, lower layer) → MeOH], and eight fractions (Fr. C-1–Fr. C-8) were yielded. Fraction C-5 (1.1 g) was separated by Sephadex LH-20 CC [MeOH–CH_2_Cl_2_ (1:1, *v*/*v*)] to gain three fractions (Fr. C-5-1–Fr. C-5-3). Fraction C-5-2 (110.0 mg) was purified by PHPLC [MeOH-H_2_O (45:55, *v*/*v*)] to afford (7*S*,8*R*)-dehydrodiconiferyl alcohol (**28**, 13.3 mg) and (–)-berchemol (**31**, 14.6 mg). Fraction C-7 (9.0 g) was isolated by ODS CC [MeOH–H_2_O (20:80 → 30:70 → 40:60 → 50:50, *v*/*v*) → MeOH], and eight fractions (Fr. C-7-1–Fr. C-7-8) were obtained. Fraction C-7-2 (351.4 mg) was purified by PHPLC [MeOH–H_2_O (32:68, *v*/*v*) + 1% HAc] to give gentiopicroside (**20**, 15.9 mg). Fraction C-7-6 (486.4 g) was subjected to Sephadex LH-20 CC [MeOH–CH_2_Cl_2_ (1:1, *v*/*v*)] and PHPLC [MeOH–H_2_O (50:50, *v*/*v*) + 1% HAc] to afford trifloroside (**17**, 16.0 mg).

Compounds **7**–**10**, **13**–**16**, **18**, **19**, **21**, **22** and **25** were obtained and identified by using the method reported previously [2,3].

*Gentiiridoside A* (**1**): White powder; [α${]}_{D}^{25}$ −90.0° (*c* 0.14, MeOH); UV *λ*_max_ (MeOH) nm (log *ε*): 229 (4.46), 285 (3.53); IR *ν*_max_ (KBr): 3372, 2929, 1712, 1635, 1588, 1486, 1372, 1264, 1201, 1154, 1078, 1017, 902, 873 cm^−1^; ^1^H-NMR (CD_3_OD, 500 MHz) and ^13^C-NMR (CD_3_OD, 125 MHz) data, see Table 1. HRESI-TOF-MS negative-ion mode *m/z* 777.2261 [M – H]^−^ (calcd for C_36_H_41_O_19_, 777.2248).

*Gentiiridoside B* (**2**): White powder; [α${]}_{D}^{25}$ −36.7° (*c* 0.12, MeOH); UV *λ*_max_ (MeOH) nm (log *ε*): 231 (4.26, sh), 273 (4.08, sh); IR *ν*_max_ (KBr) 3357, 2924, 1705, 1609, 1518, 1457, 1418, 1375, 1272, 1209, 1074, 1024 cm^−1^; ^1^H-NMR (DMSO-*d*_6_, 500 MHz) and ^13^C-NMR (DMSO-*d*_6_, 125 MHz) data see Table 2. HRESI-TOF-MS negative-ion mode *m/z* 679.1023 [M – H]^–^ (calcd for C_28_H_39_O_18_, 679.1033).

*Gentilignanoside A* (**3**): White powder; [α${]}_{D}^{25}$ −36.0° (*c* 0.10, MeOH); CD (*c* 0.0018 M, MeOH) mdeg (*λ*_nm_): −3.8 (278), −16.9 (232), −27.6 (206); UV *λ*_max_ (MeOH) nm (log *ε*): 226 (4.32), 275 (3.73); IR *ν*_max_ (KBr): 3368, 2937, 1613, 1514, 1463, 1425, 1324, 1266, 1224, 1158, 1115, 1073, 1026 cm^−1^; ^1^H-NMR (CD_3_OD, 500 MHz) and ^13^C-NMR (CD_3_OD, 125 MHz) data see Table 3. HRESI-TOF-MS negative-ion mode *m/z* 679.1023 [M – H]^−^ *m/z* 567.2083 [M – H]^−^ (calcd for C_27_H_33_O_13_, 567.2083).

*(1R)-2*,*2*,*3-Trimethyl-4-hydroxymethylcyclopent-3-ene-1-methyl-O-β-d-glucopyranoside* (**4**): White powder; [α${]}_{D}^{25}$ −43.9° (*c* 0.12, MeOH); IR *ν*_max_ (KBr): 3367, 2927, 1636, 1576, 1436, 1286, 1161, 1076, 1038 cm^−1^. ^1^H-NMR (CD_3_OD, 500 MHz) and ^13^C-NMR (CD_3_OD, 125 MHz) data see Table 4. HRESI-TOF-MS negative-ion mode *m/z* 377.1814 [M + COOH]^–^ (calcd for C_17_H_29_O_9_, 377.1817).

*(3Z)-3-Hexene-1,5-diol 1-O-α-l-arabinopyranosyl(1→6)-β-d-glucopyranoside* (**5**): White powder; [α${]}_{D}^{25}$ −14.5° (*c* 0.11, MeOH); IR *ν*_max_ (KBr): 3364, 2966, 2920, 1593, 1419, 1370, 1258, 1166, 1047, 1009 cm^−1^; ^1^H-NMR (CD_3_OD, 500 MHz) and ^13^C-NMR (CD_3_OD, 125 MHz) data see Table 5. HRESI-TOF-MS negative-ion mode *m/z* 455.1773 [M + COOH]^–^ (calcd for C_18_H_31_O_13_, 455.1770).

*Enzymatic Hydrolysis of* **4** A solution of **4** (6.0 mg) in H_2_O (2.0 mL) was reacted with β-glucosidase (6.0 mg, Almond, Sigma-Aldrich, Co. 3050 Spruce Street, St. Louis, MO, 63103 USA) at 37 °C for 2.5 h. Then the reaction mixture was extracted with EtOAc. And the residue from EtOAc solvent was subjected to Silica gel CC [CHCl_3_–MeOH (100:5, *v*/*v*)], as a result, the aglycon, (1*R*)-2,2,3-trimethyl-4-hydroxymethylcyclopent-3-ene-1-methanol (**4**a, 2.8 mg, 93.33%) was yielded.

*(1R)-2*,*2*,*3-Trimethyl-4-hydroxymethylcyclopent-3-ene-1-methanol* (**4**a): White powder; [α${]}_{D}^{25}$ −6.5° (*c* 0.09, CHCl_3_); IR *ν*_max_ (KBr): 3318, 2953, 2925, 2867, 1717, 1576, 1462, 1437, 1380, 1240, 1179, 1118, 1087, 999 cm^−1^; ^1^H-NMR (CD_3_OD, 500 MHz) and ^13^C-NMR (CD_3_OD, 125 MHz) data see Table 4. HRESI-TOF-MS negative-ion mode *m/z* 170.1423 [M – H]^−^ (calcd for C_10_H_17_O_2_, 170.1433).

*Acid Hydrolysis of* **1**–**5** The solution of compounds **1**–**5** (each 2.0 mg) in 1 M HCl (1.0 mL) was treated by using the same method as described in reference [4]: They were heated under reflux for 3 h. The reaction mixture was then analyzed by CH_3_CN–H_2_O (75:25, *v/v*; flow rate 0.7 mL/min). As results, d-glucose was detected from the aqueous phase of **1**–**5**, and l-arabinose was found from that of **5** by comparison of its retention time and optical rotation with those of the authentic sample, d-glucose (*t*_R_ 16.8 min (positive)) and l-arabinose (*t*_R_ 13.1 min (positive)), respectively.

### 3.4. Inhibitory Effects of Fractions and Compounds ***1**–**32*** on the Motility of Mouse Isolated Intestine Tissue

Inhibitory effects of fractions and compounds **1**–**32** on motility of mice isolated intestine tissue were determined by using the similar method as we reported previously [4,28]: Mice were fasted for 12 h before experiments, intestinal tissue were collected immediately. The Maxwell bath was filled with 10 mL of Tyrode’s solution (one liter contains: NaCl 8.0 g, CaCl_2_ 0.2 g, KCl 0.2 g, MgCl_2_ 0.1 g, NaHCO_3_ 1.0 g, KH_2_PO_4_ 0.05 g, glucose 1.0 g, pH 7.4) and maintained at a constant temperature (37.0 ± 0.5°C), and bubbled with 95% O_2_ and 5% CO_2_ gas. The intestinal tissue was fixed on bottom hook in and the other end was connected to an isometric tension transducer. Samples in DMSO solution were added after 15 min to equilibrate incubation, the final DMSO concentration was 0.1%, and the final concentration of fractions and compounds was 100 μg/mL and 40 μM, respectively. The mean tension and frequency of intestine muscle contractions were recorded for 1 min before and 4 min after drug additions using isolated tissue bath systems (Radnoti Glass Technology Inc., Monrovia, CA, 159901A, USA). Loperamide hydrochloride (Xi’an Janssen Pharmaceutical Ltd., Xi’an, China) was used as a positive control, and the final concentration was 10 μM.

Values are expressed as mean ± S.D. All the grouped data were statistically performed with SPSS 11.0. Significant differences between means were evaluated by one-way analysis of variance (ANOVA) and Tukey’s Studentized range test was used for post hoc evaluations. *p* < 0.05 was considered to indicate statistical significance.

## 4. Conclusions

In summary, nineteen constituents, including five new ones, gentiiridosides A (**1**), B (**2**), gentilignanoside A (**3**), (1*R*)-2,2,3-trimethyl-4-hydroxymethylcyclopent-3-ene-1-methyl-*O*-β-d-glucopyranoside (**4**), and (*3Z*)-3-hexene-1,5-diol 1-*O*-α-l-arabinopyranosyl(1→6)-β-d-glucopyranoside (**5**) were obtained from the whole plants of *G. acuta* in our on-going program of screening the phytochemical and bioactive constituents. Among the known isolates, **6**, **11**, **12**, **17**, **23**, and **27**–**32** were isolated from the genus firstly. The structures of them were elucidated by chemical and spectroscopic methods. 

Furthermore, the inhibitory effects on motility of mouse isolated intestine tissue of the above mentioned compounds and other thirteen isolates (**7**–**10**, **13**–**16**, **18**, **19**, **21**, **22** and **25**) previously obtained in the plant were analyzed. Resultingly, **5**, **7**, **10**, **12**, **14**, **16**, **17**, **31**, and **32** displayed significant inhibitory effects on contraction tension. On the other hand, structure-activity relationship analysis revealed that the inhibitory effect of secoiridoid-type monoterpenes was stronger than that of iridoid-type monoterpenes, and 7-*O*-9′-type lignan displayed stronger inhibitory bioactivity than 8-*O*-4′- and 7-*O*-4′-type lignan.

Our previous [4,5,6] and present research results suggested that the chemical constituents in *G. acuta* were xanthones, monoterpenes, lignans, and phenolic acids. Among them, xanthones and part of the monoterpenes were major bioactivity substances of *G. acuta*. These results suggested that *G. acuta* and its constituents have potential value in discovering new medicines for abnormal intestinal motility.

## Figures and Tables

**Table 1:** ^1^H and ^13^C-NMR data for **1** in CD_3_OD.

No.	δ_C_	δ_H_ (*J* in Hz)	No.	δ_C_	δ_H_ (*J* in Hz)
1	96.2	5.50 (d, 3.0)	3′′	159.2	-
3	152.4	7.46 (s)	4′′	123.5	7.42 (br. d, *ca*. 8)
4	112.8	-	5′′	131.0	7.48 (dd, 7.5, 7.5)
5	32.5	3.05 (q like, *ca*. 7)	6′′	125.1	7.84 (br. d, *ca*. 8)
6	38.0	2.00 (m)	7′′	166.1	-
		2.55 (ddd, 6.5, 7.0, 13.5)	1′′′	102.3	5.02 (d, 8.0)
7	83.7	4.96 (ddd, 4.5, 7.0, 10.5)	2′′′	77.9	3.51 (m, overlapped)
8	43.0	2.12 (m)	3′′′	74.8	3.53 (m, overlapped)
9	48.7	2.00 (m, overlapped)	4′′′	71.4	3.32 (m, overlapped)
10	18.4	1.23 (d, 7.0)	5′′′	78.1	3.33 (m, overlapped)
11	170.9	-	6′′′	62.6	3.66 (dd, 4.0, 11.5)
1′	100.2	4.69 (d, 7.5)			3.84 (br. d, *ca*. 12)
2′	74.6	3.22 (dd, 7.5, 8.5)	1′′′′	133.1	-
3′	77.8	3.40 (dd, 8.5, 8.5)	2′′′′	123.9	7.82 (br. s)
4′	71.3	3.46 (dd, 8.5, 8.5)	3′′′′	152.3	-
5′	78.2	3.51 (m, overlapped)	4′′′′	127.7	7.45 (br. d, *ca*. 8)
6′	62.4	3.75 (dd, 5.0, 11.5)	5′′′′	130.8	7.54 (dd, 7.5, 7.5)
		3.92 (br. d, *ca*. 12)	6′′′′	128.1	7.88 (br. d, *ca*. 8)
1′′	131.7	-	7′′′′	166.8	-
2′′	119.3	7.89 (br. s)			

**Table 2:** ^1^H and ^13^C-NMR data for **2** in DMSO-*d*_6_.

No.	δ_C_	δ_H_ (*J* in Hz)	No.	δ_C_	δ_H_ (*J* in Hz)
1	96.4	5.59 (d, 3.0)	6′	61.0	3.44 (m, overlapped)
3	148.8	7.41 (s)			3.68 (br. d, *ca*. 12)
4	103.2	-	1′′	96.8	4.27 (d, 7.5)
5	124.9	-	2′′	74.7	2.90 (dd, 7.5, 8.5)
6	116.1	5.65 (m)	3′′	76.6	3.12 (m, overlapped)
7	69.1	4.97 (dd, 3.0, 18.0)	4′′	70.2	3.05 (m, overlapped)
		5.04 (br. d, *ca*. 18)	5′′	76.7	3.03 (m, overlapped)
8	134.0	5.72 (ddd, 6.5, 10.5, 17.5)	6′′	61.1	3.44 (m, overlapped)
9	44.3	3.31 (m)			3.66 (br. d, *ca*. 12)
10	117.9	5.21 (m)	1′′′	92.1	4.91 (d, 3.5)
11	162.7	-	2′′′	72.3	3.12 (m, overlapped)
1′	98.7	4.49 (d, 8.0)	3′′′	72.7	3.42 (m, overlapped)
2′	73.0	2.95 (dd, 8.0, 8.5)	4′′′	70.5	3.05 (m, overlapped)
3′	76.7	3.15 (m, overlapped)	5′′′	71.9	3.57 (m)
4′	69.9	3.03 (m, overlapped)	6′′′	61.1	3.44 (m, overlapped)
5′	77.3	3.15 (m, overlapped)			

**Table 3:** ^1^H and ^13^C-NMR data for **3** in CD_3_OD.

No.	δ_C_	δ_H_ (*J* in Hz)	No.	δ_C_	δ_H_ (*J* in Hz)
1	129.9	-	7′	35.1	2.54 (dd, 12.5, 12.5)
2,6	106.2	6.64 (s)			3.13 (br. d, *ca*. 13)
3,5	148.9	-	8′	51.8	2.59 (m)
4	136.2	-	9′	72.0	3.64 (m, overlapped)
7	85.8	4.84 (s)			4.06 (dd, 7.5, 7.5)
8	83.3	-	3,5-OCH_3_	56.8	3.84 (s)
9	64.6	3.64 (m, overlapped)	3′-OCH_3_	56.8	3.86 (s)
		3.80 (m, overlapped)	1′′	103.1	4.88 (d, 7.5)
1′	136.9	-	2′′	75.0	3.49 (dd, 7.5, 8.5)
2′	114.4	6.90 (br. s)	3′′	77.9	3.45 (m)
3′	150.9	-	4′′	71.4	3.40 (m, overlapped)
4′	146.5	-	5′′	78.2	3.40 (m, overlapped)
5′	118.3	7.10 (d, 8.0)	6′′	62.6	3.68 (dd, 5.0, 11.5)
6′	122.4	6.77 (br. d, *ca*. 8)			3.89 (m, overlapped)

**Table 4:** ^1^H and ^13^C-NMR data for **4** and **4a** in CD_3_OD.

	4		4a	
No.	δ_C_	δ_H_ (*J* in Hz)	δ_C_	δ_H_ (*J* in Hz)
1	49.0	2.15 (m, overlapped)	51.7	2.00 (m)
2	49.3	-	49.0	-
3	143.5	-	143.7	-
4	133.1	-	133.2	-
5α	36.6	2.15 (dd, 8.0, 9.0)	36.5	2.09 (dd, 8.0, 9.0)
5β		2.48 (dd, 8.0, 8.0)		2.49 (dd, 8.0, 8.0)
1-CH_2_OH	72.1	3.66 (m, overlapped)	64.2	3.54 (dd, 8.5, 11.0)
		3.95 (dd, 6.5, 11.0)		3.72 (dd, 6.5, 11.0)
2α-CH_3_	27.2	1.09 (s)	27.3	1.08 (s)
2β-CH_3_	20.1	0.87 (s)	19.9	0.85 (s)
3-CH_3_	9.4	1.56 (s)	9.4	1.56 (s)
4-CH_2_OH	59.4	4.07 (m)	59.4	4.06, 4.10 (both d, 12.0)
1′	104.5	4.26 (d, 7.5)		
2′	75.2	3.17 (dd, 7.5, 8.0)		
3′	78.3	3.34 (dd, 8.0, 8.0)		
4′	71.7	3.29 (m, overlapped)		
5′	78.0	3.29 (m, overlapped)		
6′	62.8	3.66 (m, overlapped)		
		3.87 (br. d, *ca*. 12)		

**Table 5:** ^1^H and ^13^C-NMR data for **5** in CD_3_OD.

No.	δ_C_	δ_H_ (*J* in Hz)	No.	δ_C_	δ_H_ (*J* in Hz)
1	70.4	3.61 (dd, 6.5, 11.5)	4′	71.7	3.33 (dd, 8.0, 8.0)
		3.86 (m, overlapped)	5′	76.9	3.43 (m)
2	29.3	2.39 (m)	6′	69.6	3.72 (dd, 5.5, 11.0)
		2.46 (m)			4.09 (dd, 2.0, 11.0)
3	127.4	5.45 (m)	1′′	105.2	4.31 (d, 6.5)
4	137.0	5.47 (m)	2′′	72.4	3.58 (dd, 6.5, 9.0)
5	64.4	4.61 (m)	3′′	74.2	3.52 (dd, 3.5, 9.0)
6	23.9	1.20 (d, 6.0)	4′′	69.5	3.80 (m)
1′	104.4	4.27 (d, 7.5)	5′′	66.7	3.53 (dd, 2.0, 12.5)
2′	75.1	3.17 (dd, 7.5, 8.0)			3.86 (m, overlapped)
3′	78.0	3.34 (dd, 8.0, 8.0)			

**Table 6:** Inhibitory effects of fractions and compounds **1**–**32** on motility of mouse isolated intestine tissue.

	Intestine Motility (%)			Intestine Motility (%)	
	Relative Tension	Relative Frequency		Relative Tension	Relative Frequency
N	100.0 ± 4.0	100.0 ± 7.3	**14**	90.1 ± 2.6 *	102.1 ± 1.9
P	74.1 ± 9.3 *	82.7 ± 5.3 *	**15**	88.9 ± 5.0	104.7 ± 2.3
A	83.1 ± 3.4 *	97.5 ± 3.6	**16**	80.5 ± 6.1 *	102.1 ± 5.8
B	93.9 ± 4.2	103.2 ± 3.1	**17**	64.9 ± 7.1 **	94.3 ± 2.0
C	74.5 ± 3.8 ***	99.3 ± 5.3	**18**	88.2 ± 4.9	86.4 ± 8.8
D	78.4 ± 3.5 ***	103.2 ± 4.4	**19**	93.7 ± 7.1	100.3 ± 2.4
**1**	93.2 ± 3.5	102.0 ± 3.7	**20**	85.8 ± 5.0	98.2 ± 1.4
**2**	88.2 ± 3.4	97.7 ± 4.9	**21**	86.8 ± 4.6	102.2 ± 3.2
**3**	88.8 ± 5.1	113.0 ± 14.0	**22**	89.6 ± 4.4	112.0 ± 8.1
**4**	100.4 ± 2.0	100.4 ± 2.0	**23**	91.2 ± 2.7	103.6 ± 7.4
**5**	81.6 ± 2.9 **	98.5 ± 0.9	**24**	91.0 ± 3.5	101.0 ± 4.4
**6**	89.5 ± 7.0	100.9 ± 5.5	**25**	91.0 ± 3.8	102.1 ± 2.6
**7**	82.9 ± 7.0 *	100.8 ± 2.6	**26**	84.8 ± 4.7	105.9 ± 1.6
**8**	86.6 ± 4.6	85.2 ± 3.0	**27**	77.4 ± 7.4	100.8 ± 5.6
**9**	91.8 ± 3.3	100.7 ± 2.3	**28**	76.7 ± 10.1	97.4 ± 3.5
**10**	75.5 ± 6.4 **	99.7 ± 1.4	**29**	90.4 ± 9.0	102.6 ± 1.0
**11**	88.9 ± 5.0	98.4 ± 3.3	**30**	91.0 ± 6.3	95.9 ± 3.3
**12**	75.7 ± 9.1 *	96.6 ± 4.6	**31**	79.1 ± 3.7 **	102.3 ± 5.2
**13**	84.9 ± 4.0 *	100.3 ± 1.4	**32**	82.4 ± 4.7 *	92.2 ± 1.3

Values are the means ± standard error of measurement, significantly different from the control group, * *p* < 0.05, ** *p* < 0.01, *** *p* < 0.001, *n* = 6. Normal (N): isolated intestine tissue; Positive control (P): Loperamide hydrochloride, final concentration was 10 μM. A: *G. acuta* 70% EtOH extract; B: H_2_O eluate from D101 resin for extract; C: 95% EtOH eluate from D101 resin for extract; D: CHCl_3_ layer for extract, and their final concentration was 100 μg/mL. Compounds **1**–**32**: final concentration was 40 μM. Tension and frequency of normal group was set as 100%, relative tension, and frequency were calculated as: (sample/normal) × 100%.
